# Health-Related Quality of Life, Treatment Satisfaction, Adherence and Persistence in **β**-Thalassemia and Myelodysplastic Syndrome Patients with Iron Overload Receiving Deferasirox: Results from the EPIC Clinical Trial

**DOI:** 10.1155/2012/297641

**Published:** 2012-08-12

**Authors:** John Porter, Donald K. Bowden, Marina Economou, Jacques Troncy, Arnold Ganser, Dany Habr, Nicolas Martin, Adam Gater, Diana Rofail, Linda Abetz-Webb, Helen Lau, Maria Domenica Cappellini

**Affiliations:** ^1^Department of Haematology, UCL Cancer Institute, University College London, Paul O'Gorman Building, 72 Huntley Street, London WC1E 6BT, UK; ^2^Monash Medical Centre, Melbourne, VIC 3168, Australia; ^3^Thalassemia Clinical Care Services Unit, Hippokration General Hospital Thessaloniki, Egnatia Street 106, 54622 Thessaloniki, Greece; ^4^Hematology, Hopital Edouard Herriot, 6 Rue Antoine Lumiere, 69008 Lyon, France; ^5^Medizinische Hochschule Hannover (MHH), Department of Hematology, Hemostasis, Oncology and Stem Cell Transplantation, Carl-Neuberg Strasse 1, 30625 Hannover, Germany; ^6^Novartis Pharmaceutical Corporation, 180 Park Avenue, 105-3E065, Florham Park, NJ 07932-1080, USA; ^7^Novartis Pharma AG Postfach, 4002 Basel, Switzerland; ^8^Adelphi Values, Adelphi Mill, Grimshaw Lane, Bollington, Cheshire SK10 5JB, UK; ^9^Universita di Milano, Can Granda Foundation IRCCS, Via F. Sforza 35, 20122 Milan, Italy

## Abstract

Treatment of iron overload using deferoxamine (DFO) is associated with significant deficits in patients' health-related quality of life (HRQOL) and low treatment satisfaction. The current article presents patient-reported HRQOL, satisfaction, adherence, and persistence data from **β**-thalassemia (*n* = 274) and myelodysplastic syndrome (MDS) patients (*n* = 168) patients participating in the Evaluation of Patients' Iron Chelation with Exjade (EPIC) study (NCT00171821); a large-scale 1-year, phase IIIb study investigating the efficacy and safety of the once-daily oral iron chelator, deferasirox. HRQOL and satisfaction, adherence, and persistence to iron chelation therapy (ICT) data were collected at baseline and end of study using the Medical Outcomes Short-Form 36-item Health Survey (SF-36v2) and the Satisfaction with ICT Questionnaire (SICT). Compared to age-matched norms, **β**-thalassemia and MDS patients reported lower SF-36 domain scores at baseline. Low levels of treatment satisfaction, adherence, and persistence were also observed. HRQOL improved following treatment with deferasirox, particularly among **β**-thalassemia patients. Furthermore, patients reported high levels of satisfaction with deferasirox at end of study and greater ICT adherence, and persistence. Findings suggest deferasirox improves HRQOL, treatment satisfaction, adherence, and persistence with ICT in **β**-thalassemia and MDS patients. Improving such outcomes is an important long-term goal for patients with iron overload.

## 1. Introduction

Regular blood transfusions are essential for the management of haematological conditions such as *β*-thalassemia major and myelodysplastic syndromes (MDS). As a result, however, patients with these conditions are susceptible to the development of transfusion-dependent iron overload (hemosiderosis or secondary iron overload). In the absence of a naturally occurring physiological mechanism for the removal of excess iron in the body, life-long treatment and adherence to iron chelation therapy (ICT) are necessary to prevent the morbidity and mortality that may result if excess iron is allowed to accumulate [1, 2]. 

Deferoxamine (DFO), most commonly delivered by continuous subcutaneous infusion over 8 to 12 hours a day, is the oldest available form of ICT used by patients with transfusion-dependent disorders. Prior research, albeit in small sample sizes, has indicated significant deficits in health-related quality of life (HRQOL) among patients receiving DFO for the treatment of transfusion-dependent iron overload, compared to values from age-matched normative populations [3, 4]. In particular, the time-consuming nature of DFO regimens and side effects associated with this form of ICT (including local site reactions) [5–7] can have a detrimental impact on numerous facets of patients' lives, including work; social activities; sex life; sleep; emotional well-being [8]. As a result, patient satisfaction with DFO treatment regimens is low and suboptimal adherence is common among patients [3, 4]. Improvements in ICT administration convenience and tolerability are expected to improve patient's satisfaction with ICT and HRQOL, thus promoting adherence to ICT regimens and potentially reducing iron overload-related morbidity/mortality and associated healthcare costs [1, 9, 10]. 

Deferasirox (Exjade) is an oral ICT first approved in 2005 and is the most widely prescribed ICT today [11]. Deferasirox has been shown to be an efficacious and generally well-tolerated therapy for the treatment of iron overload in *β*-thalassemia and MDS patients [12, 13]. Findings from randomised control trials comparing outcomes in patients with iron overload treated using either deferasirox or DFO have also suggested the superiority of deferasirox in terms of treatment satisfaction and adherence [14, 15]. However, additional research using validated patient-reported outcome (PRO) measures is needed in order to better understand the added benefits of deferasirox over DFO in terms of reducing HRQOL burden and improving treatment satisfaction, adherence, and persistence among patients with transfusion-dependent iron overload. The current work seeks to address these needs by presenting and discussing PRO findings from the Evaluation of Patients' Iron Chelation with Exjade (EPIC) study (NCT00171821), a large-scale prospective study designed to investigate the efficacy and safety of deferasirox in patients diagnosed with transfusion-dependent iron overload [12, 13].

## 2. Methods

### 2.1. Study Design

The EPIC study was a prospective, 1-year, multicentre, open-label phase IIIb trial conducted by 136 investigators across 23 countries [12, 13]. A PRO substudy within the EPIC trial was conducted to assess self-reported HRQOL and treatment satisfaction, adherence, and persistence in patients with transfusion-dependent iron overload. Based on the availability of validated questionnaire translations, the PRO substudy included participating study sites in Australia, Belgium, France, Germany, Greece, Italy, the Netherlands, and the UK. Study findings reported here focus specifically on data from adult patients (≥16 years of age) with *β*-thalassemia and MDS. Based on the inherent differences in the underlying disease and patient profiles, study findings for patients with *β*-thalassemia and MDS will be reported separately. Of note, however, PRO data were also collected from patients with a variety of other transfusion-dependent disorders (including sickle cell disease, aplastic anemia, and other rare anemias) but sample sizes were considered too small (*n* < 30) for evaluable analysis as separate subgroups.

In accordance with EPIC trial selection criteria, all patients enrolled in this study were required to have transfusion-related iron overload, evident by a serum ferritin level of ≥1000 ng/mL or with LIC > 2 mg Fe/g dw, as determined by R2-Magnetic Resonance Imaging (MRI) [12, 13]. Patients unsuitable for participation in a clinical study from a clinical perspective (e.g., presence of systemic diseases which would prevent the patient from undergoing treatment) or a practical perspective (e.g., history of non-compliance to medical regimens) were excluded from the study. Assessments of HRQOL and satisfaction, adherence, and persistence to ICT were collected at baseline and at the end of the study (EOS; week 52 or at time of early study discontinuation), where appropriate, using the Medical Outcomes Short-Form 36-item Health Survey (SF-36v2) and the Satisfaction with ICT Questionnaire (SICT). Questionnaires were provided to patients in the native language of the respective country in which the patient was enrolled. Both questionnaires have been linguistically validated for use in the respective countries, ensuring the cross-cultural equivalence of the questionnaires and enabling data collected from different countries to be considered in a single-pooled dataset.

### 2.2. Ethics

The EPIC study was conducted in accordance with the Declaration of Helsinki; the International Conference on Harmonization (ICH) Tripartite Guidelines for Good Clinical Practice 1996; the Rules Governing Medicinal Products in the European Community (Directive 91/507/EEC); the US 21 Code of Federal Regulations dealing with clinical studies. Written informed consent was obtained from all patients prior to participation in the PRO substudy.

### 2.3. PRO Measures

#### 2.3.1. The Medical Outcomes Short-Form 36-Item Health Survey (SF-36v2)

The SF-36v2 is a self-administered questionnaire comprising 36-items measuring eight dimensions of general HRQOL: physical functioning (10 items), role limitation due to physical health problems (4 items), bodily pain (2 items), general health perceptions (5 items), vitality (4 items), social functioning (2 items), role limitations due to emotional problems (3 items), and general mental health (5 items). In addition to scores for individual dimensions, two summary scores assessing physical and mental dimensions of health and well-being can also be calculated: Physical Component Summary (PCS) score and the Mental Component Summary (MCS) score, respectively. 

Although specific “tools” for the assessment of HRQOL have been developed for thalassemia [16], the SF-36 has the advantage of having been used extensively within clinical trials and academic studies, across a wide range of disease areas, including *β*-thalassemia and MDS [3, 17–20]. The psychometric validity and reliability of the instrument as a generic measure of health-related functional status and well-being is well established [21–23]. In this study, the SF-36v2 was collected from patients at both baseline and EOS. All data were handled and scored in accordance with the developer's instructions: item scores for each dimension were coded, summed, and transformed to a scale from 0 (worst possible health state) to 100 (best possible health state), whereby higher values indicate better HRQOL. Domain scores were only calculated if at least half of all items comprising a domain were completed by the patient; missing data was not imputed [22]. 

#### 2.3.2. Satisfaction with ICT Questionnaire (SICT)

The SICT is a questionnaire designed specifically to assess patient satisfaction with ICT regimens [24]. It comprises 19 items assessing four domains of patient satisfaction: perceived effectiveness of ICT (PE), burden of ICT (BD), acceptance of ICT (AC), and side effects of ICT (SE). Patients rate all items on a response scale from 1 “very dissatisfied” to 5 “very satisfied”. Domain scores are calculated as the mean score across constituent items and a higher score indicates greater satisfaction with respect to the questionnaire domain. As with the SF-36v2, domain scores were calculated if at least half of all items comprising a domain were completed by the patient; no missing data was imputed [24].

In addition, the SICT also includes three individual items designed to assess adherence to ICT “How often did you follow the chelation therapy regimen exactly as recommended by your doctor?”, ICT persistence “How often did you think about stopping your chelation therapy?”, and difficulties remembering to take ICT “How often did you have trouble remembering to take your chelation therapy?”. All three items are assessed on a 5-point Likert scale from 1 “Always” to 5 “Never” and are designed to be interpreted as standalone items of the respective concepts. 

Previous studies in patients with a variety of transfusion-dependent haematological disorders have provided evidence that the SICT is a reliable and valid measure of iron overload patients' satisfaction, adherence, and persistence to ICT regimens [24, 25]. All patients participating in the PRO substudy completed the SICT at EOS. Only patients with prior history of ICT were required to complete the SICT at baseline; the SICT was not relevant at this timepoint for those patients with no prior history of ICT. 

#### 2.3.3. Statistical Analyses and Data Interpretation

Descriptive statistics for subscale domains and summary component scores of the SF-36v2 were computed at baseline and EOS. To highlight the HRQOL burden associated with iron overload, mean SF-36 domain, and summary scores at baseline, and EOS were compared to published data of patients with *β*-thalassemia or MDS and age-matched norms derived from the UK general population [3, 17, 22, 26]. Confidence interval estimates were used to evaluate the significance of differences in observed study means relative to other reference groups. SICT domain scores and responses to questions regarding patient-reported adherence and persistence with ICT therapy utilization were also summarized at baseline and EOS. 

Relevant differences in group means between study and other reference populations for SF-36 domain scores (e.g., disease-specific and UK general population) were evaluated using a distribution-based approach for establishing clinically meaningful difference. In this regard, differences that are 0.5 standard deviation (SD) units of a baseline score were characterized as clinically meaningful [27–29]. 

Analysis of questionnaires at baseline and EOS (e.g., week 52 or at time of early study discontinuation) was undertaken only in cases where sample sizes were large enough (*n* > 30) for statistical analyses. Data were presented separately for patients with underlying *β*-thalassemia and MDS due to inherent differences in disease populations and patient profiles.

## 3. Results

### 3.1. Demographic and Clinical Characteristics

The demographic and clinical characteristics of *β*-thalassemia (*n* = 274) and MDS (*n* = 168) patients evaluated in this PRO substudy are displayed in Table 1 and are generally similar to those of the overall *β*-thalassemia and MDS populations enrolled in the EPIC trial [12, 13]. As expected, the mean age of patients with MDS was considerably higher than the mean age of patients with *β*-thalassemia. Almost all *β*-thalassemia patients (*n* = 270; 98.5%) had a history of prior ICT, with 66.4% (*n* = 184) having previously received DFO monotherapy and 30.3% (*n* = 84) having previously received DFO and deferiprone. Only 51.8% (*n* = 87) of MDS patients had a history of prior ICT, however, with 37.5% (*n* = 63) of the MDS sample having previously received DFO monotherapy and 8.3% (*n* = 14) having previously received DFO and deferiprone. As such, where relevant, differences between MDS patients with a history of ICT and ICT-naïve patients are highlighted. Note, however, that sample sizes of evaluable data do not allow for statistical comparison of differences between these two groups.

### 3.2. Changes in HRQOL following Treatment with Deferasirox

#### 3.2.1. *β*-Thalassemia Patients

**Figure 1:**
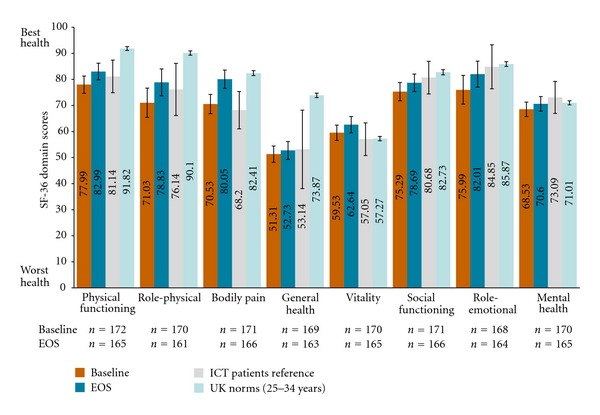
Mean SF-36 domain scores for *β*-thalassemia population versus disease-related and general population references at baseline and EOS. Data illustrated in the above figure are representative of the mean and 95% confidence interval estimates of the mean as calculated using the formula: 1.96 ∗ standard deviation $÷\sqrt{n}$. Reference data for ICT patients were based on Payne et al. [3] (where 82% of patients in this study were thalassemia patients); UK norms from Jenkinson et al. [42] and direct email communications from Dr. Jenkinson on Oct 24, 2011 with age-specific norms.

At baseline, mean scores for 6 of the 8 SF-36 domains among patients with *β*-thalassemia were notably lower than equivalent scores derived from UK norms for persons aged 25 to 34 years (the exceptions being vitality and mental health). Of these domains, differences in scores for physical functioning, role-physical, and general health at baseline among patients with *β*-thalassemia, compared to UK general population norms, were at a level considered to be clinically meaningful; indicating significant burden within this population. However, baseline SF-36 domain scores among these patients were similar to historical reference patients previously receiving infused chelation therapy as reported by Payne et al. and in which 82% of patients were *β*-thalassemia patients [3] (Figure 1). 

Mean SF-36 PCS and MCS scores for *β*-thalassemia patients at baseline ($\overset{-}{x}=45.64$ [SD = 9.25] and $\overset{-}{x}=47.72$  [SD = 10.63], resp.) were also lower compared to UK norms (by −6.94 and −0.35 points, resp.); however, only the PCS score was substantially different and considered clinically meaningful. Relative to other patients with a history of receiving infusional ICT, mean summary component scores at baseline for patients with *β*-thalassemia in the EPIC study were similar.

Mean SF-36 domain scores at EOS were generally higher following treatment with deferasirox and closer to population norm scores for the UK general population and patients previously receiving infusional chelation therapy [3]. Meaningful changes in scores from baseline to EOS were observed for bodily pain, role-physical, and role-emotional in patients with *β*-thalassemia. Mean SF-36 domain scores at EOS were lower compared to UK population norms for physical functioning, role-physical and general health domains, but only the general health domain score at EOS was lower than population norms to a clinically meaningful degree. Mean SF-36 PCS and MCS scores were also higher at EOS compared to baseline ($\overset{-}{x}=48.15$  [SD = 9.03] and $\overset{-}{x}=48.67$  [SD = 9.52], resp.). PCS scores were, however, still substantially lower than population norms such that the difference can be considered clinically meaningful. 

#### 3.2.2. MDS Patients

**Figure 2:**
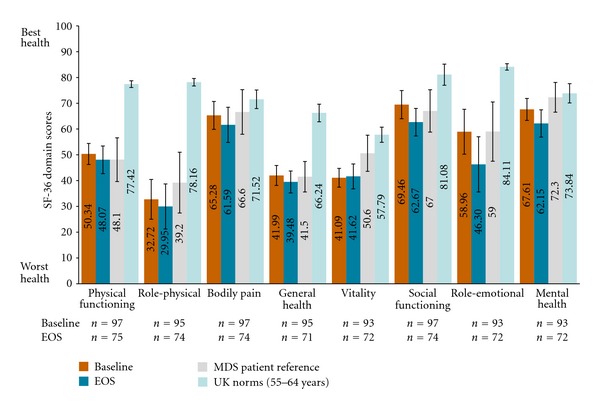
Mean SF-36 domain scores for MDS population versus MDS and general population references at baseline and EOS. Data illustrated in the above figure are representative of the mean and 95% confidence interval estimates of the mean as calculated using the formula: 1.96 ∗ standard deviation $÷\sqrt{n}$. Transfusion-dependent MDS patient reference data reported by Jansen et al. [17]; UK norms from Jenkinson et al. [42] and direct email communications from Dr. Jenkinson on Oct 24, 2011 with age-specific norms.

In patients with MDS, mean baseline scores for all SF-36 domains were lower than age-matched UK norms for persons aged 65–74 years but were similar to other patients with MDS (Figure 2) as previously reported by Jansen et al. [17]. Differences between MDS and the UK normative sample were also at the level considered to be clinically meaningful for physical functioning, role-physical, general health, vitality, social functioning, and role-emotional domains. Mean SF-36 PCS and MCS scores for MDS patients at baseline ($\overset{-}{x}=35.47$  [SD = 8.58] and $\overset{-}{x}=47.16$  [SD = 11.29], resp.) were also lower than those derived from the UK normative sample (−9.00 and −5.12, resp.), however, only differences in PCS were of a clinically meaningful magnitude. Relative to other MDS patients as reported by Jansen et al. (2003), mean summary component scores at baseline for patients with MDS in the EPIC study were similar [17].

In patients with MDS, mean SF-36 domain scores were lower at EOS compared to age-matched UK population norms for all SF-36 domain scores. Mean scores at EOS were no different from means of MDS patients as reported by Jansen et al. [17]. Except for the bodily pain domain, all other functional and well-being domains of the SF-36 scale were lower than UK norms to a clinically meaningful degree. Compared to baseline, PCS scores remained relatively stable ($\overset{-}{x}=35.71$  [SD = 9.64]), but MCS scores declined at EOS ($\overset{-}{x}=43.56$  [SD = 11.79]). At EOS, deficits for both PCS and MCS were lower than UK norms to a substantial and clinically meaningful degree. 

Further analysis of SF-36 scores for MDS patients with and without prior ICT history suggest that ICT-naïve patients had at baseline lower PCS scores and lower domain scores for physical functioning, role-physical, bodily pain, and general health domains, compared to patients with prior experience of ICT. However, ICT naïve patients had higher MCS scores and scores for all constituent domains, except for vitality (Table 2). Scores for all SF-36 domains and summary components were lower at EOS compared to baseline among patients with prior history of ICT. SF-36 domains for ICT-naïve patients were higher for physical functioning, role-physical, general health, vitality, and PCS but were lower for other domains and MCS at EOS. 

### 3.3. Changes in ICT Satisfaction following Treatment with Deferasirox

#### 3.3.1. *β*-Thalassemia Patients

**Figure 3:**
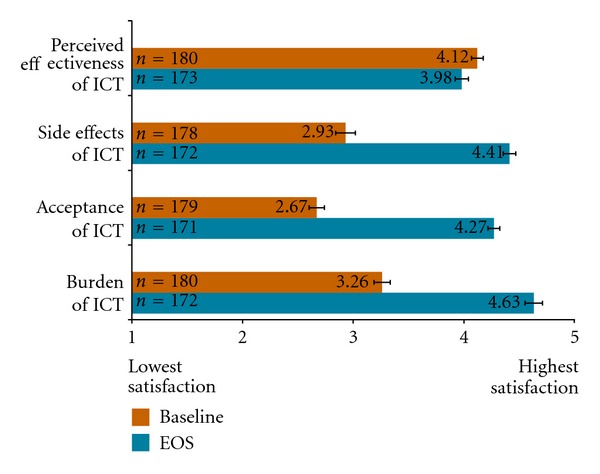
Mean SICT domain scores at baseline and EOS for overall *β*-Thalassemia population.

At baseline, patients with *β*-thalassemia and prior history of ICT were generally satisfied with the perceived effectiveness of their ICT prior to initiation of deferasirox in the EPIC study but were neither satisfied nor dissatisfied with side effects of ICT, acceptance of ICT and burden of ICT as measured by SICT domains (Figure 3). Compared to baseline, patient-reported satisfaction associated with side effects of ICT, acceptance of ICT, and burden of ICT SICT domains increased with deferasirox by ≥1.4 points at EOS. 

#### 3.3.2. MDS Patients

**Figure 4:**
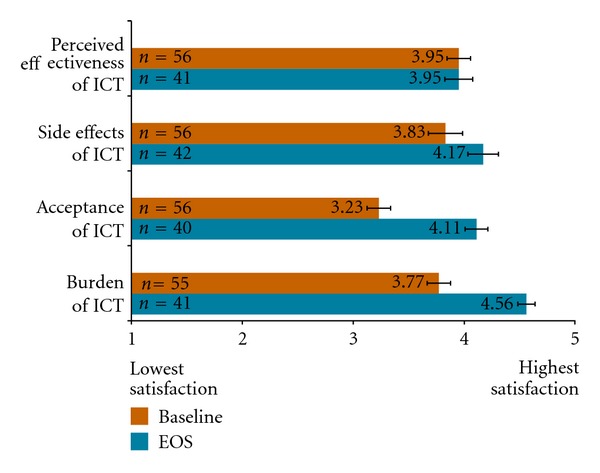
Mean SICT domain scores at baseline and EOS for MDS population with prior history of ICT.

At baseline, patient-reported satisfaction with ICT was high (>3.5) among MDS patients with prior history of ICT as measured by SICT domains of perceived effectiveness of ICT, side effects of ICT, and burden of ICT (Figure 4). Patients were neither satisfied or dissatisfied on SICT domain of acceptance of ICT at baseline. Compared to baseline, patient-reported satisfaction with ICT-related side effects, acceptance, and burden were higher at EOS following treatment with deferasirox based on SICT domain scores. Scores for the perceived effectiveness of ICT SICT domain remained stable between baseline and EOS. 

SICT data were also evaluated at EOS for those MDS patients with no prior history of ICT. Notably, the SICT domain scores reported were comparable to EOS scores for patients with prior ICT for SICT domains: side effects of ICT ($\overset{-}{x}=4.18$, SD = 0.17), acceptance of ICT ($\overset{-}{x}=4.17$, SD = 0.69), and burden of ICT ($\overset{-}{x}=4.51$, SD = 0.55). Patient-reported satisfaction for the perceived effectiveness of ICT domain was also high at EOS ($\overset{-}{x}=4.25$, SD = 0.60).

### 3.4. Changes in ICT Adherence following Treatment with Deferasirox

Following treatment with deferasirox, the proportion of *β*-thalassemia patients who reported always following their ICT regimen as recommended by their doctor increased from 32.4% (*n* = 58/179) at baseline to 67.1% (*n* = 116/173) at EOS. Patient-reported adherence was also high among patients with MDS who had a prior history of ICT at baseline where 62.5% (*n* = 35/56) of patients reported having always followed their ICT regimen as they were told by their doctor. Following treatment with deferasirox, patient's self-reported adherence to ICT increased to 85.7% (*n* = 36/42) at EOS. Similarly, patient-reported adherence among MDS patients with no prior history of ICT at EOS was high, with 82.9% (*n* = 29/35) of patients reporting that they always followed their ICT regimen as they were told by their doctor.

### 3.5. Changes in ICT Persistence following Treatment with Deferasirox

Following treatment with deferasirox in the EPIC study, the proportion of patients with *β*-thalassemia who never thought about stopping ICT increased from 40.8% (*n* = 73/179) at baseline to 76.3% (*n* = 132/173) at EOS. Among patients with MDS and a prior history of ICT, the proportion of patients who never thought about stopping ICT was high at baseline (75.9%,  *n* = 41/54), but decreased slightly at EOS following treatment with deferasirox (69.0%,  *n* = 29/42). Persistence to ICT, however, was high at EOS among MDS patients with no prior history of ICT; 77.1% (*n* = 27/35) of this population never thought about stopping ICT at EOS. 

## 4. Discussion

The EPIC study is the largest prospective evaluation of any iron chelation therapy conducted to date. Findings from this study have demonstrated that deferasirox is an efficacious and generally well-tolerated treatment for the treatment of iron overload in patients with transfusion-dependent disorders such as *β*-thalassemia and MDS [12, 13]. In addition, this study provided a unique opportunity to collect data concerning the role that deferasirox can play in addressing HRQOL concerns associated with iron chelation therapies and how deferasirox may help address issues related to treatment satisfaction, adherence, and persistence among patients with iron overload.

SF-36 data collected for *β*-thalassemia patients at baseline suggest that these patients have lower HRQOL compared to age-matched norms on almost all facets of HRQOL assessed within the SF-36. The greatest deficits were seen in related “physical” domains of the SF-36 (e.g., physical functioning, role-physical, bodily pain, and general health). These findings are supportive of prior investigations among smaller samples of *β*-thalassemia patients and confirm assertions that *β*-thalassemia is a debilitating disease [3, 4, 18]. Similarly, as reflective of prior research, deficits on all facets of HRQOL were also observed among patients with MDS [17, 30]. Notably, however, HRQOL deficits appeared greater among patients with MDS compared to those with *β*-thalassemia. 

The burdensome nature of infused ICT (DFO) has been cited as a contributory factor to diminished HRQOL domains observed in patients with transfusion-dependent disorders [3, 4, 8]. As such there is an expectation that, compared to DFO, oral ICT such as deferasirox will have a less detrimental effect on patient's HRQOL. Although the EPIC study does not provide a direct head-to-head comparison of deferasirox versus DFO, the prospective evaluation of HRQOL over time following initiation of deferasirox provides unique insight into the differential impact of both infused and oral ICT on HRQOL among patients with *β*-thalassemia and MDS. This is especially true for *β*-thalassemia patients, many of whom had been receiving DFO prior to enrolment in the EPIC study. The prospective assessment of HRQOL over time in MDS patients also addresses the limitations of prior research in MDS patients which, in collecting HRQOL data at one time point, give little indication of changes in HRQOL in this population over time. 

Observations from the EPIC study suggest that deferasirox was associated with directional improvements in all facets of HRQOL as assessed by the SF-36 among patients with *β*-thalassemia which were similar to prior reported studies of similar populations [3, 17]. Of most note were improvements in bodily pain; a key but often overlooked symptom of *β*-thalassemia [31]. In contrast to patients with *β*-thalassemia, patients with MDS in the EPIC study had slightly lower mean HRQOL domain scores at EOS, however, mean scores were similar to observations reported by Jansen et al. [17] and may be representative of declining prognosis and disease progression with transfusion dependence and the need for supportive care [32, 33]. 

Observed differences in HRQOL between the two populations must be interpreted in the context of the demographic and clinical characteristics of the respective populations. In contrast to the *β*-thalassemia which is a genetic condition typically diagnosed in early years of life, MDS is an acquired disorder with the majority of cases occurring in patients over the age of 60 [34]; a difference reflected in the substudy samples for the EPIC trial (mean age of 26 versus 68 years, resp.). In addition, modern-day therapy has increased life expectancy for *β*-thalassemia patients, such that patients can now live for decades [35, 36]. Past research has also indicated that HRQOL in patients with *β*-thalassemia remains relatively stable over time; adding confidence that changes observed in this study are a result of the study treatment (e.g., switch to deferasirox) as opposed to statistical artifact [37]. By contrast, the prognosis of patients with MDS is generally poor due to disease progression, deterioration, and increasing transfusion dependence [38]. The presence of comorbid conditions (e.g., diabetes, coronary heart disease, or chronic pulmonary obstructive disease) which are increasingly prevalent among elderly populations also complicate the management of MDS and contribute to poor risk among these patients [39]. As such, HRQOL may be expected to deteriorate among MDS populations over time, independent of the form of ICT that is received by the patient. 

The EPIC study provides unique insight into the impact that deferasirox may have on patients HRQOL over a one-year period. However, the key to minimising long-term morbidity and mortality in patients with transfusion-dependent disorders is ensuring patient's adherence to recommended ICT regimens. The burdensome nature of infusional ICT regimens or iterative ICT combination regimens may complicate compliance and result in suboptimal adherence, and persistence [3, 4]. This was also evident in the present study, particularly among patients with *β*-thalassemia where approximately 40% of patients at baseline self-reported that they always followed their treatment regimen as recommended by their doctor (e.g., adherence) and never thought about stopping ICT treatment (e.g., persistence). However, self-reported adherence and persistence among patients with *β*-thalassemia increased at EOS following treatment with deferasirox (67.1% and 76.3%, resp.). Likewise, the proportion of MDS patients with prior history of ICT who reported always following their ICT regimen as recommended by their doctor increased at EOS compared to baseline. Self-reported persistence with ICT was slightly lower at EOS compared to baseline (75.9% and 69.0% never thought about stopping their ICT at baseline and EOS, resp.) among MDS patients with prior history of ICT which may be associated with underlying disease progression. 

Patients who are less satisfied with prescribed treatment are expected to be less likely to adhere to recommended treatment protocols. Consistent with this, patients with *β*-thalassemia in the EPIC study demonstrated a higher level of satisfaction with ICT at EOS following treatment with deferasirox, particularly in relation to practical aspects of ICT such as side-effects, burden, and acceptability of treatment regimen which coincided with higher self-reported adherence and persistence with deferasirox at EOS. In MDS patients with prior history of ICT, notable improvements in satisfaction with ICT were recorded between baseline and EOS and higher self-reported adherence. This observation suggests that the directional changes in HRQOL in patients with MDS may be associated with other factors such as progressive underlying disease, complications of older age, among others, and the may not be related to study treatment. Furthermore, levels of satisfaction among patients with MDS and no prior history of ICT were also high at EOS, offering further support that the directional changes in aspects of HRQOL between baseline and EOS may be attributable to other factors.

In reflecting on potential limitations of the present study, the number of MDS patients for whom there was evaluable data should be considered. The high rate of study discontinuations among MDS patients participating in the EPIC study is acknowledged as source of potential bias in these data and is a key factor limiting the availability of evaluable data in this substudy. As detailed by Gattermann et al. (2010), of 341 patients with MDS enrolled, 175 patients completed the study (median duration of treatment: 50.6 weeks) whereas 166 discontinued (rate: 48.7%) with the primary reason for study discontinuation being due to adverse events (*n* = 78) [13]. Nonetheless, when interpreting findings from the current study, it is necessary to consider that HRQOL, treatment satisfaction, adherence, and persistence may be negatively affected in those patients discontinuing treatment due to adverse events. 

In interpreting the findings of this study, it is also important to appreciate that the defining features of controlled clinical studies (e.g., study selection criteria, predetermined assessment schedules, study treatment) may introduce bias to the evaluation of health outcomes (e.g., HRQOL, treatment satisfaction, adherence, persistence, etc.). In particular, the exclusion of participants with a history of poor compliance to medical regimens may have affected ratings of adherence, and persistence at baseline and EOS. As such, further consideration of naturalistic studies would help to establish the real-world validity of observed changes in HRQOL, treatment satisfaction, adherence and persistence associated with deferasirox observed in the EPIC study. A recent retrospective assessment of iron chelation adherence from the Thalassemia Clinical Research Network (TRCN) also documents high patient-reported adherence with deferasirox [40, 41]. With this end in mind, the present study provides further evidence of the validity of the SF-36 and SICT as measures of HRQOL and treatment satisfaction/adherence/persistence, respectively, and supports their use in future studies of transfusion-dependent conditions and ICTs. Standardisation of PRO assessments in future studies (using the SF-36 and SICT, e.g.) is particularly important for enabling meaningful comparisons to be made between varying ICT regimens. 

## 5. Conclusions

This substudy represents the largest prospective evaluation of patient-reported outcomes with deferasirox to date. Findings indicate improvements in patient-reported HRQOL, ICT satisfaction, adherence, and persistence following treatment with deferasirox particularly among *β*-thalassemia patients, the majority of whom had been using infused ICT prior to enrolment in the study. Patient satisfaction with deferasirox is high and patients receiving deferasirox report being more likely to adhere and persist with ICT. Such evaluations are vital for improving both the long-term health outcomes and survival of patients with transfusion-dependent iron overload and minimising future health resource use.

## Figures and Tables

**Table 1:** Demographic and clinical characteristics of *β*-thalassemia and MDS patients.

	*β*-thalassemia (*N* = 274)	MDS (*N* = 168)
Mean age (SD)	26 (11.5)	68 (10.3)
Males, *n* (%)	127 (46.4)	96 (57.1)
Age groups, *n* (%)		
<6 yrs	17 (6.2)	0 (0)
6 to <12 yrs	25 (9.1)	0 (0)
12 to <16 yrs	15 (5.5)	0 (0)
16 to <50 yrs	213 (77.7)	4 (2.4)
50 to <65 yrs	4 (1.5)	55 (32.7)
≥65 yrs	0 (0)	109 (64.9)
Prior chelation therapy, *n* (%)		
No	4 (1.5)	81 (48.2)
Yes	270 (98.5)	87 (51.8)
Prior chelation therapy, *n* (%)		
None	4 (1.5)	81 (48.2)
DFO only	184 (66.4)	63 (37.5)
Deferiprone only	2 (0.7)	9 (5.4)
DFO and deferiprone^∗^	84 (30.3)	14 (8.3)
Other ICT	3 (1.1)	1 (0.6)

^∗^Patients may have received both DFO and deferiprone as prior chelation therapies, but these may not have been in combination.

**Table 2:** Mean (SD) SF-36 domain and summary scores among MDS patients according to prior ICT history.

			Baseline mean (SD)		EOS mean (SD)	
	Reference MDS population (*n* = 50): mean (SD)	Age-matched UK norms: mean (SD)	Prior ICT (*N* = 59–62)	ICT Naïve (*N* = 34-35)	Prior ICT (*N* = 38–40)	ICT Naïve (*N* = 32–35)
Physical functioning	48.1	77.42	53.60	44.56	47.43	48.79
	(30.6)	(25.38)	(21.35)	(17.94)	(24.39)	(23.29)
Role-physical	39.2	78.16	36.39	26.43	30.21	29.66
	(42.5)	(28.11)	(39.21)	(36.85)	(40.90)	(35.60)
Bodily pain	66.6	71.52	65.61	64.69	63.23	59.77
	(31.2)	(26.46)	(27.43)	(27.16)	(28.53)	(31.85)
General health	41.5	66.24	42.70	40.72	37.78	41.55
	(21.3)	(22.57)	(18.94)	(20.00)	(20.01)	(15.92)
Vitality	50.6	57.79	43.25	37.35	42.89	40.20
	(25.3)	(21.28)	(19.02)	(15.92)	(24.10)	(16.81)
Social functioning	67.0	81.08	68.55	71.07	62.18	63.21
	(29.6)	(26.14)	(29.75)	(23.24)	(25.57)	(21.21)
Role-emotional	59.0	84.11	57.18	61.90	41.23	51.96
	(41.4)	(24.41)	(44.05)	(41.34)	(43.45)	(45.83)
Mental health	72.3	73.84	66.78	69.06	60.07	64.47
	(20.9)	(19.35)	(22.34)	(18.54)	(22.85)	(23.01)
Physical component summary	35.7	44.47	36.97	33.10	36.21	35.11
	(11.7)	(12.32)	(8.48)	(8.31)	(8.70)	(10.80)
Mental component summary	48.9	52.28	46.32	48.49	42.14	45.27
	(12.6)	(9.89)	(12.02)	(10.04)	(11.61)	(11.97)

Transfusion-dependent MDS patient reference data reported by Jansen et al. [17]; UK norms from Jenkinson et al. [42] and direct email communications from Dr. Jenkinson on Oct 24, 2011 with age-specific norms.
